# Targeting Anion Exchange of Osteoclast, a New Strategy for Preventing Wear Particles Induced- Osteolysis

**DOI:** 10.3389/fphar.2018.01291

**Published:** 2018-11-06

**Authors:** Chuanlong Wu, Xuqiang Liu, Ruixin Sun, Yunhao Qin, Zhiqing Liu, Shengbing Yang, Tingting Tang, Zhenan Zhu, Degang Yu, Fengxiang Liu

**Affiliations:** ^1^Shanghai Key Laboratory of Orthopaedic Implants, Department of Orthopaedics, Ninth People’s Hospital, Shanghai Jiao Tong University School of Medicine, Shanghai, China; ^2^Department of Orthopaedics, Ruijin Hospital, Shanghai Jiao Tong University School of Medicine, Shanghai, China; ^3^Department of Orthopaedics, The First Affiliated Hospital, Nanchang University, Nanchang, China; ^4^State Key Laboratory of Oncogenes and Related Genes, Shanghai Cancer Institute, Renji Hospital, Shanghai Jiao Tong University School of Medicine, Shanghai, China; ^5^Department of Orthopaedics, Sixth People’s Hospital, Shanghai Jiao Tong University, Shanghai, China

**Keywords:** bone resorption, wear particle, osteoclast, SLC4A2, actin

## Abstract

Joint replacement is essential for the treatment of serious joint disease. However, prosthetic failure remains an important clinical issue, with periprosthesis osteolysis (PO), caused by osteoclastic bone resorption induced by wear particles, being the leading cause of failure. Nuclear factor of activated T cells c1 (NFATc1) appears to play an important role in wear particle-induced osteoclastogenesis, with bicarbonate/chloride exchanger, solute carrier family 4, anion exchanger, member 2, (SLC4A2) being upregulated during osteoclastogenesis in an NFATc1-dependent manner. Anion exchange mediated by SLC4A2 in osteoclasts could affect the bone resorption activity by regulating pHi. This study investigated the role and mechanism of SLC4A2 in wear particle-induced osteoclast differentiation and function *in vitro*. The use of 4, 4′-diisothiocyano-2,2′-stilbenedisulfonic acid (DIDS), an anion exchange inhibitor, suppressed wear particle-induced PO *in vivo*. Furthermore, controlled release of DIDS from chitosan microspheres can strengthen the PO therapy effect. Therefore, anion exchange mediated by osteoclastic SLC4A2 may be a potential therapeutic target for the treatment of aseptic loosening of artificial joints.

## Introduction

Periprosthetic osteolysis (PO) and subsequent aseptic loosening remains a major problem for the long-term success and survival of prosthetic joints (Yang et al., 2004). Osteolysis induced by wear debris is an important concern considering that wear particles appear in tissues around a prosthesis in 70–90% cases (Baumann et al., 2004). Specifically, wear particles promote osteoclast differentiation and activation, which leads to bone loss around the prosthesis, accompanied by an increase in peripheral inflammatory mediators and dysfunction of osteoblasts, with the process eventually leading to PO (Goodman and Ma, 2010). As the degradation of material, due to movement between the prosthetic components, is unavoidable, then preventing the activation of osteoclasts, which is the direct cause of PO, could provide a therapeutic target to prevent PO and aseptic loosening of joint prostheses (Broomfield et al., 2017; Sartori et al., 2017).

Osteoclasts are multinucleated giant cells that differentiate from myeloid precursors (Segovia-Silvestre et al., 2009) under the influence of osteoblast-derived cytokines, the macrophage colony-stimulating factor (MCSF) and the receptor activator for nuclear factor-B ligand (RANKL). This process is controlled by the nuclear factor of activated T cells c1 (NFATc1) which, itself, is induced by RANKL and governs the expression of genes necessary for osteoclast formation and function (Asagiri and Takayanagi, 2007; Takayanagi, 2007; Aliprantis and Glimcher, 2010). In a previous study (Liu et al., 2009), we demonstrated the expressions of NFATc1 in bone marrow monocytes (BMMs) and multinucleated cells cultured with titanium (Ti) particles and RANKL. Inactivation of NFATc1 by the 11R-VIVIT peptide could provide a potent inhibition of Ti particle-induced osteoclastogenesis. However, the NFATc1 pathway is active in various cell reaction processes and, therefore, its inhibition may cause numerous side effects, including immunity suppression (Muller and Rao, 2010; Sitara and Aliprantis, 2010; Fric et al., 2012). Thus, the downstream pathway of NFATc1 is important to consider as a therapeutic target.

Bicarbonate/chloride exchanger, solute carrier family 4, anion exchanger, member 2 (*Slc4a2*), the main gene of anion exchanger, is upregulated during osteoclast differentiation in an NFATc1-dependent manner (Wu et al., 2008). Excessive bone resorbing activity of osteoclasts is the direct reason for wear-particle-induced osteolysis (Greenfield et al., 2002). Bone resorption begins with the differentiation of macrophage precursors into osteoclasts, which adhere to the bone surface, polarizing the surface to form an extracellular hemivacuole into which acid is secreted (Teitelbaum, 2000). The secretion of protons into this resorption lacuna generates an intracellular acid deficit (Teitelbaum, 2000). SLC4A2 is located on the basolateral membrane of osteoclasts and is responsible for the uptake of HCO_3_^-^ by osteoclasts (Wu et al., 2008). Previous studies showed that SLC4A2-mediated anion exchanger could affect the bone resorption activity by regulating pHi in osteoclast, thus, anion exchange inhibitors could repress bone resorption (Rousselle and Heymann, 2002; Supanchart and Kornak, 2008).

Therefore, targeting SLC4A2-mediated- anion exchange of osteoclast may be a new strategy for preventing wear particle-induced osteolysis. To our knowledge, there is little information regarding this field. Therefore, our aim in this study was to clarify this putative role of SLC4A2-mediated anion exchange in PO.

## Materials and Methods

### Reagents

Fetal bovine serum (FBS) and alpha modification of Eagle’s medium (α-MEM) were supplied by Gibco-BRL (Sydney, Australia). Bacteria-derived recombinant mouse RANKL and soluble human recombinant M-CSF were purchased from R&D Systems (Minneapolis, MN, United States). 4,4′-diisothiocyano-2,2′-stilbenedisulfonic acid (DIDS) and the Diagnostic Acid Phosphatase kits for tartrate-resistant acid phosphatase (TRAP) staining were supplied by Sigma. The 11R-VIVIT peptide (RRRRRRRRRRR-GGG-MAGPHPVIVITGPHEE) was obtained from Sigma Genosys (Woodlands, TX, United States).

### Wear Particles Preparation

Commercial pure Ti particles (average diameter: 4.50 μm) were supplied by Johnson Matthey (Ward Hill, MA, United States), and the commercial polymethylmethacrylate (PMMA) particles (average diameter: 6.0 μm) were obtained from Polyscience Inc. (Warrington, PA, United States). Ti and PMMA particles were prepared as previously reported to remove endotoxins (Liu et al., 2009, 2013). Briefly, Ti particles were sterilized by baking at 180°C for 6 h, followed by treatment with 70% (v/v) ethanol for 48 h, and PMMA particles were treated with 70% (v/v) ethanol for 48 h. Such particles have been shown to effectively mimic wear particles retrieved from periprosthetic tissues (Lee et al., 2003; von Knoch et al., 2004).

### Bone Marrow-Derived Macrophages (BMM)’ Isolation and Osteoclast Culture

Primary mouse BMM cells were isolated from the long bones of 5-week-old C57BL/6 mice and cultured in α-MEM containing 10% heat inactivated FBS, 2 mM L-glutamine, 100 U/mL penicillin/streptomycin, and 30 ng/mL M-CSF (complete α-MEM medium), as described previously (Wu et al., 2015). These cells were cultured at 37°C, in a humid environment with 5% CO_2_. RANKL and M-CSF are necessary factors to induce BMM cell differentiation into osteoclasts (Darnay et al., 1998; Wada et al., 2006). Therefore, we used both RANKL and M-CSF for induction. BMM cells were cultured in a complete α-MEM medium, with or without RANKL (100 ng/mL), with or without 0.1 mg/mL Ti/PMMA particles, with or without other special treatments.

### RNA Interference, Adenoviral Expression Vectors and Infection

Short hairpin RNAs (shRNAs) were designed and synthesized by Invitrogen. BMM cells were transfected at ∼50% confluence with shRNAs, using Lipofectamine RNAiMAX (Thermo Fisher Scientific, Waltham, MA, United States). The sequences for successful shRNAs were given in Tables 1, 2.

**Table 1:** shRNA of *Slc4a2.*

Gene		Initiation site
shRNA 1	5′-GCTATGGAGAGGAAGACTTTG-3′	262
shRNA 2	5′-GCCCAAGTCTGCCCAAGATA-3′	2014
shRNA 3	5′-GCAATGAGTTGGAGTACTTGG-3′	2410

**Table 2:** *Slc4a2* shRNA primers.

Gene	Forward	Reverse
shRNA 1	GATCCGCTAT GGAGAGGAA GACTTTGT TCAAGAGA CAAAGTCTTCC TCTCCATAGCAGA	GCGATAT CCCTCCT TCTGAAACAA GTTCTCT GTTTCAG AAGGAGAGGTA TCGTCTTCGA
shRNA 2	GATCCGCCCAA GTCTGCCCAA GATAATT CAAGAGATTA TCTTGGGCAG ACTTGGGCAGA	GCGGGTTCA GACGGGTTC TATTAAGT TCTCT AATA GAACCCGTCT GAACC CGTCTTCGA
shRNA 3	GATCCGCAAT GAGTTGG AGTACTT GGCTTCAAGAGA CCAAGTACTCCAA CTCATTGCAGA	GCGTTAC TCAACCTCAT GAACCAAGT TCTCT GGTTCATGAGGT TGAGT AACGTC TTCGA

### RNA Isolation and Reverse Transcription Quantitative PCR (RT-qPCR)

RT-qPCR was used to measure the expression of *Slc4a2*, *Nfatc1*, *Trap*, cathepsin K (*Ctsk*), calcitonin receptor (*Ctr*), and *Gapdh* mRNAs. BMM cells were seeded in 6-well plates at a density of 1 × 10^5^ cells/well and cultured in complete α-MEM. During RANKL-induced osteoclastogenesis, the BMMs were treated with or without 100 ng/mL RANKL, with or without 0.1 mg/mL Ti/PMMA particles, with or without shRNAs pre-treatments, and with or without 11R-VIVIT (2/5/10 μM). After the culture was sustained for 0–5 days, RT-PCR was used to assess the expression of these genes’ mRNAs in BMMs. The method of RT-qPCR in detail was described in a previous study (Wu et al., 2015). Total RNA was extracted using the Qiagen RNeasy Mini kit (Qiagen, Valencia, CA, United States) in accordance with the manufacturer’s instructions, and cDNA was synthesized from 1 mg of total RNA using reverse transcriptase (TaKaRa). Real-time PCR was performed using SYBR1 Premix Ex Taq^TM^ II (TaKaRa) and an ABI 7500 Sequencing Detection System (Applied Biosystems, Foster City, CA, United States). The following cycling conditions were used: 40 cycles of denaturation at 95°C for 5 s and amplification at 60°C for 24 s. GAPDH was amplified as a housekeeping gene, and all reactions were run in triplicate. The sequences of the RT-qPCR primers were as follow:

*Slc4a2* forward 5′-GTGCAGAAAGGAAGCCAGAG-3′ and
reverse 5′-TCTTCGCTCCTGAAGGTTGT-3′;
*Nfatc1* forward 5′-CCGTTGCTTCCAGAAAATAACA-3′ and
reverse 5′-TGTGGGATGTGAACTCGGAA-3′;
*Trap* forward 5′-CTGGAGTGCACGATGCCAGCGACA-3′ and
reverse 5′-TCCGTGCTCGGCGATGGACCAGA-3′;
*Ctsk* forward 5′-CTTCCAATACGTGCAGCAGA-3′ and
reverse 5′-TCTTCAGGGCTTTCTCGTTC-3′;
*Ctr* forward 5′-TGCAGACAACTCTTGGTTGG-3′ and
reverse 5′-TCGGTTTCTTCTCCTCTGGA-3′;
*Gapdh* forward 5′-ACCCAGAAGACTGTGGATGG-3′ and
reverse 5′-CACATTGGGGGTAGGAACAC-3′.

### TRAP Staining of Osteoclasts

Bone marrow monocyte cells were seeded into a 96-well plate, at a density of 1 × 10^4^ cells/well, in a complete α-MEM medium, with RANKL (100 ng/mL), with or without 0.1 mg/mL Ti/PMMA particles, and with or without shRNA3 pre-treatment. The culture medium was replaced every 2 days until osteoclast formation occurred in the negative control group. The cells were fixed with 4% paraformaldehyde for 20 min and stained for TRAP using the Diagnostic Acid Phosphatase kit. The area of TRAP-positive cells was counted by ImageJ software (National Institutes of Health).

### Bone Pit Resorption Assay

Bone marrow monocyte cells were seeded directly onto ivory slices and cultured in a complete α-MEM medium, with RANKL (100 ng/mL), with or without 0.1 mg/mL Ti/PMMA particles, with or without shRNA3 pre-treatment, until osteoclast-like (OCL) cells were observed in the negative control group. The OCL cells were then treated and resorption pits were visualized using a scanning electron microscope (SEM, FEI Quanta 250) as per a previous study (Wu et al., 2015). ImageJ software was used to quantify the percentage of resorbed bone surface area.

### Immunofluorescence Staining

Bone marrow monocytes were cultured in complete α-MEM medium with RANKL (100 ng/mL), with or without 0.1 mg/mL Ti/PMMA particles, with or without shRNA3 pre-treatment, until OCL cells were observed in the negative control group. The F-actin ring of OCL cells were dyed with rhodamine-conjugated phalloidin (1:100; Invitrogen Life Technologies, United States), examined using a NIKON A1Si spectral detector confocal system equipped with a 20 (dry) lenses, collected using NIS-C Elements software and analyzed using ImageJ software; details were described in a previous study (Wu et al., 2015).

### Preparation of Chitosan Microspheres (CMs)

Chitosan microspheres (CMs) were prepared through the water-in-oil (W/O) emulsion solvent diffusion method. A specific amount of chitosan (CS, 25 mg) with medium molecular weight was dissolved through sonication in 1% (w/v) acetic acid solution to make up chitosan concentrations at 0.20% (w/v) and the pH was adjusted to 5.0. DIDS powder, which was accurately weighed, was dissolved in water to prepare a concentration of 25 mg/ml solution. The prepared DIDS solution was then slowly dropped into the CS solution using a micro-syringe. Finally, 2.5 ml of sodium tripolyphosphate solution having a concentration of 2.0 mg/ml was added to the above mixture under mechanical stirring (600 rpm). The reaction mixture was kept for 1.5 h at room temperature. Chitosan nanoparticles (CS-NPs) without DIDS were prepared by the same method. The controlled release capacity of the CMs was determined by measuring the optical density (OD) value of the extracts at 342 nm (which is specific for DIDS) (Hua and Inesi, 1997). The standard curve for DIDS and the control release curve for the CMs was made using OriginPro 8 SRO (Northampton, MA, United States).

### Wear Particle-Induced Calvarial Osteolysis Model

As described previously, we established a mouse calvarial osteolysis model and used this to measure the osteolysis-suppressing effect of DIDs *in vivo* (Liu et al., 2014). This study obtained ethics approval by the Animal Care Committee of Shanghai Jiao Tong University. All experimental procedures and welfare related assessment were performed in accordance with the guidelines of the Animal Care Committee of Shanghai Jiao Tong University. Seventy-two healthy 8-week-old male c57BL/J6 mice, weighing 22 ± 1 g, were purchased from Shanghai SLAC Laboratory Animal Co., Ltd. All animals were housed under conditions of constant temperature (20–26°C) and humidity (40–70%) on a 12-h light/dark cycle. Food and water were available *ad libitum*. Plastic lab rodent mice cages were used and six mice in one cage. We anesthetized mice with ketamine and xylazine and sacrificed them with carbon dioxide euthanasia. The mice we used all are male and weighing 22 ± 1 g. So the mice were grouped according to body-weight randomly using Excel.

Forty-two healthy 8-week-old C57BL/J6 mice were assigned randomly to seven groups: PBS control (sham), Ti particles (Ti vehicle), Ti particles with low (2 mg/kg/day) and high (5 mg/kg/day) doses of DIDS, PMMA particles (PMMA vehicle), and PMMA particles with low and high DIDS. In each group, PBS/or 30 mg of Ti/PMMA particles were embedded under the periosteum at the middle suture of the calvaria (Qin et al., 2012) and at 2 days after implantation, DIDS or PBS was injected into the periosteum every other day for 14 days. No adverse effects or mortality occurred during the experiment. At the end of the experiment, the mice were sacrificed and the calvaria were excised (Figure 4A). 3D reconstruction was conducted through micro-computed tomography (CT) scanning as reported previously (Liu et al., 2014). Additionally, hematoxylin and eosin (HE) and TRAP staining were conducted by histological sections, and the area of TRAP-positive multinucleated osteoclasts was determined for each sample (Liu et al., 2014).

Furthermore, the method of effect of controlled release of DIDS from CMs on wear particle-induced PO was the same as described above. Thirty healthy 8-week-old C57BL/J6 mice were assigned randomly to five groups: PBS control (sham), Ti particles (Ti vehicle + CM), Ti particles with CM + DIDS (14 mg/kg/once), PMMA particles (PMMA vehicle + CM), and PMMA particles with CM + DIDS (14 mg/kg/once). At 2 days after wear particle implantation, CM + DIDS or PBS was injected into the periosteum once (Figure 5D). 3D reconstruction, bone volume/tissue volume (BV/TV), and the percentage of total porosity of each sample were measured through micro-computed tomography (CT) scanning as reported previously (Liu et al., 2014).

### Statistical Analysis

Data are expressed as the mean ± standard error of measurement (SEM). Differences between groups were analyzed using analysis of variance (ANOVA). At least three independent replicated of each experiment were conducted separately. Statistical significance was defined as a *P*-value < 0.05. Statistical analyses were performed using SPSS (version 11.0; Chicago, IL, United States).

## Results

### *Slc4a2* Plays an Important Role During Wear Particle-Induced Osteoclastogenesis

**FIGURE 1:**
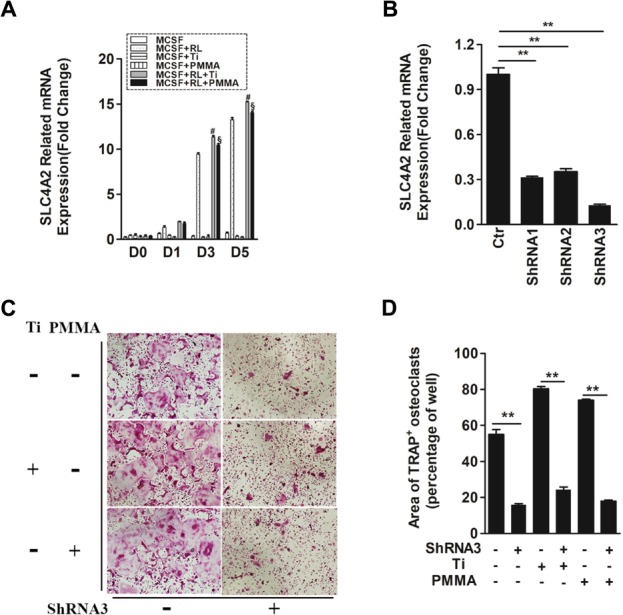
SLC4A2 plays an important role in wear particle-induced osteoclastogenesis. **(A)** Up-regulation of *Slc4a2* gene expression in the process of wear particle-induced osteoclastogenesis. Bone marrow-derived macrophages (BMM) were used [#, MCSF+RANKL(RL)+Ti vs. MCSF+RL, *P* < 0.01; §, MCSF+RL+PMMA vs. MCSF+RL, *P* < 0.01]. **(B)** Three different shRNA vectors targeting *Slc4a2*, with shRNA3 yielding the greatest reduction in *Slc4a2* mRNA. Effective knockdown of *Slc4a2* in BMM cells, at 48 h after transfection using *Slc4a2* shRNA1, shRNA2 and shRNA3, respectively. After transfection, cells were induced to differentiate into osteoclasts and harvested to examine *Slc4a2* expression using reverse transcription quantitative PCR (RT-qPCR). **(C)** Effect of knockdown of *Slc4a2*, using shRNA3, on wear particle-induced osteoclastogenesis *in vitro*. **(D)** The area of TRAP-positive cells, measured using ImageJ (^∗^*P* < 0.05; ^∗∗^*P* < 0.01). At least three independent replicated of each experiment were conducted separately.

*Slc4a2* is the main gene of anion exchanger in osteoclasts (Wu et al., 2008). Thus, we wanted to know the expression of *Slc4a2* in wear particle-induced osteoclastogenesis. Bone marrow-derived macrophages (BMMs) were used. *Slc4a2* gene expression in mouse osteoclast precursor cells is low. In the absence of RANKL, wear particles failed to induce *Slc4a2* gene expression, which is consistent with a stimulation of osteoclast precursors to osteoclasts by wear particles (Figure 1A). This finding suggests that *Slc4a2* gene expression is dependent on osteoclast differentiation.

To evaluate the possibility of aberrant *Slc4a2* activation in wear particle-induced osteoclastogenesis, we compared *Slc4a2* expression using RT-qPCR analysis in Ti- or PMMA-induced osteoclastogenesis and normal osteoclastogenesis. Across all groups, there was little-to-no expression of *Slc4a2* on day 0. In the absence of RANKL, *Slc4a2* expression remained low up to day 5. By comparison, in the control group, expression of the *Slc4a2* gene increased during the process of Ti- and PMMA particle-induced osteoclast differentiation (Figure 1A). There was almost no *Slc4a2*expression detected on day 0 after Ti or PMMA particles were added to the BMM cells, with little increase after 1 day. However, expression of the *Slc4a2*gene significantly increased on day 3 and day 5. Although this trend was also observed in the control group, *Slc4a2* gene expression was higher in the particle-induced group (day 3/5, *P* < 0.01) (Figure 1A).

Evidence for the pathological role of SLC4A2 in osteoclast prompted us to analyze the functional relevance of SLC4A2 inhibition to the development of wear particle-induced osteoclastogenesis. First, three different shRNA vectors targeting *Slc4a2* were designed. RT-qPCR results showed that shRNA3 produced the most effective knockdown of *Slc4a2* in BMM cells (Figure 1B). Secondly, we investigated whether *Slc4a2* inhibition prevented wear particle induced-osteoclastogenesis *in vitro*. The results of TRAP staining showed that osteoclast precursor cells were induced to mature to osteoclasts in the control and Ti particle group but with larger osteoclasts formed in the Ti particle-induced group. The knockdown of *Slc4a2* expression significantly inhibited Ti particles-induced osteoclast differentiation, as well as osteoclast differentiation in the control group (*p* < 0.01) (Figures 1C,D).

We also observed the effect of blocking *Slc4a2* expression on PMMA particle-induced osteoclast differentiation. Similar to the above results, PMMA particle induced osteoclast precursor cells were also induced to differentiate into osteoclasts. Knockdown of *Slc4a2* expression by shRNA3 decreased the formation of TRAP-positive multinucleated osteoclasts (*p* < 0.01) (Figures 1C,D).

Collectively, these results confirmed that suppression of *Slc4a2* expression inhibited wear particle-induced osteoclastogenesis *in vitro*.

### Suppression of *Slc4a2* Expression in BMM Cells Inhibited Wear Particle-Induced Osteoclast Function *in vitro*

**FIGURE 2:**
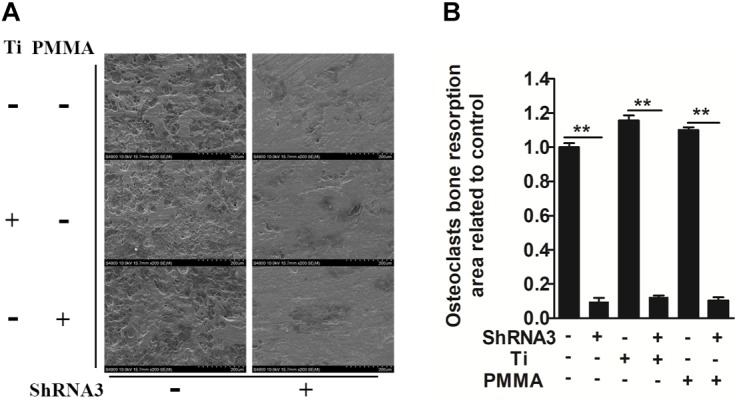
Role of SLC4A2 in wear particle-induced osteoclast function. Equal numbers of osteoclasts were seeded onto bone slices, allowed to adhere to the surface, and treated with 30 ng/mL M-CSF, 100 ng/mL RANKL, and the indicated treatment. **(A)** Knockdown of SLC4A2, using shRNA3, on wear particle-induced osteoclastic bone resorption *in vitro*. Representative scanning electron microscopy (SEM) images of bone-resorption pits are shown. **(B)** The total area of resorption pits, measured using ImageJ (^∗^*P* < 0.05; ^∗∗^*P* < 0.01). At least three independent replicated of each experiment were conducted separately.

We investigated whether *Slc4a2* inhibition prevents wear particle induced-osteoclast bone resorption function *in vitro*. SEM results showed that obvious bone resorption lacunae were formed on the bone surface, with a clear boundary and a round, oval or sausage-shaped morphology, in the control group. RNA interference (shRNA3) significantly decreased the prevalence of bone resorption lacunae (*P* < 0.01, Figures 2A,B). Collectively, these results confirmed that suppression of *Slc4a2* expression inhibited the wear particle-induced osteoclast bone resorption function *in vitro*.

### SLC4A2 Regulates Wear Particle-Induced Pathological Changes in BMM Cells Partially Through Its Effect on the Actin Cytoskeleton

**FIGURE 3:**
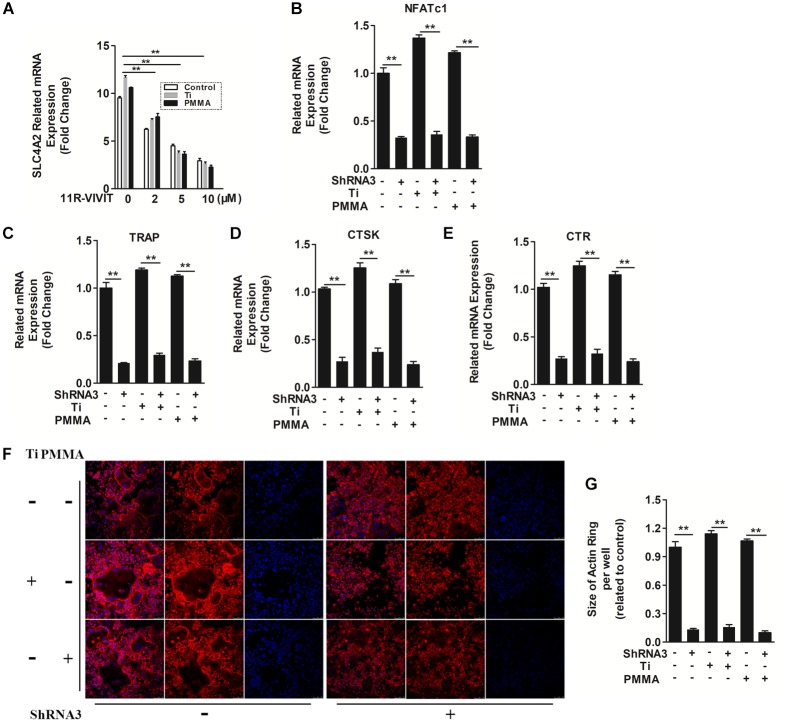
The mechanism of SLC4A2 in wear particle-induced osteoclast differentiation and function. **(A)** 11R-VIVIT, an inhibitor of NFATc1 suppressed up-regulation of *Slc4a2* expression during wear particle-induced osteoclastogenesis. **(B)** Expression of *Nfatc1* was inhibited by knockdown of *Slc4a2* with shRNA3 unexpectedly. **(C–E)** Suppression of osteoclast-specific gene expression (*Trap*, *Ctsk*, *Ctr*) with knockdown of *Slc4a2* with shRNA3. **(F)** Knockdown of *Slc4a2*, using shRNA3, inhibits F-actin ring formation *in vitro*. **(G)** The size of F-actin rings, measured using ImageJ (^∗^*P* < 0.05; ^∗∗^*P* < 0.01). At least three independent replicated of each experiment were conducted separately.

SLC4A2 is an NFATc1-regulated transcript in osteoclasts, with its mutation blocking skeletal remodeling (Wu et al., 2008). 11R-VIVIT, an inhibitor of the NFATc1-activating phosphatase calcineurin (Liu et al., 2009), attenuated *Slc4a2* expression in a dose dependent manner (Figure 3A). Thus, *Slc4a2* is upregulated during wear particle induced-osteoclastogenesis in an *Nfatc1*-dependent manner. On the contrary, suppression of *Slc4a2* expression also inhibited wear particle-induced *Nfatc1* upregulated expression, which indicated that these signal pathways may function in a loop (Figure 3B).

At the same time, the expression of several genes is upregulated during osteoclast differentiation. We used RT-qPCR to analyze the effect of suppressing *Slc4a2* expression on RANKL-induced mRNA expression of osteoclast-related genes. Wear particles potently strengthen the expression of all the evaluated genes (*Trap*, *Ctr*, and *Ctsk*) (Liu et al., 2009). However, the wear particle and RANKL-induced upregulation of these genes was strongly suppressed in the presence of shRNA3 (*P* < 0.01, Figures 3C–E). Collectively, these results confirmed that suppression of *Slc4a2* expression inhibited wear particle and RANKL-induced osteoclasts special gene expression *in vitro*.

In light of these data, we set out to uncover the underlying mechanisms of how SLC4A2 regulates wear particle-induced pathological changes in BMM cells. SLC4A2 regulates reorganization of the actin cytoskeleton, via an effect on anion-exchange activity, and intracellular pH (Coury et al., 2013). Confocal microscopy revealed a characteristic complete and clear actin cytoskeleton ring formation in control osteoclasts stained with phalloidin-Alexa Fluor 647. The actin ring size of osteoclasts seems to be larger when wear particles (Ti or PMMA) were added. However, the knockdown of SLC4A2 with shRNA3 drastically disrupted actin ring formation and size (Figures 3F,G). These data showed that SLC4A2 regulates wear particle-induced pathological changes in BMM cells partially through its effect on the actin cytoskeleton.

### Administration of DIDS, an Anion Exchange Inhibitor, Prevented Wear-Particle-Induced Bone Loss *in vivo*

**FIGURE 4:**
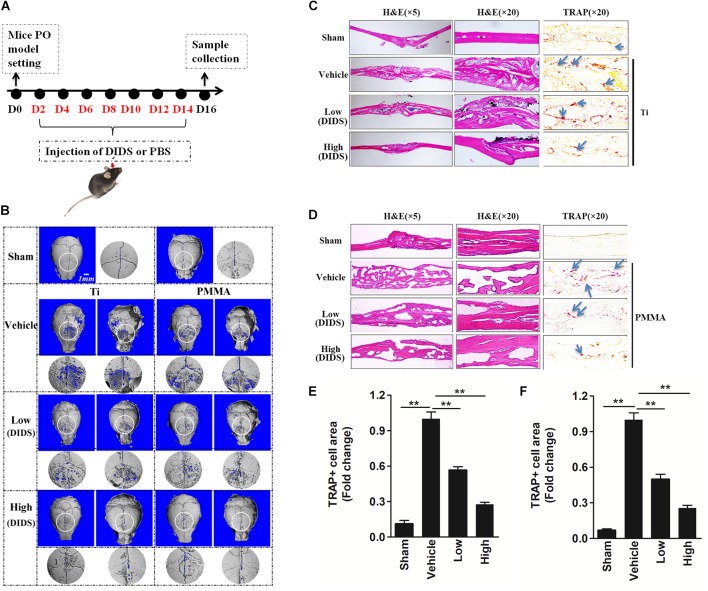
DIDS, an anion exchange inhibitor, prevented wear-particle-induced osteolysis in a mouse calvarial model. **(A)** Timeline of the experiment. **(B)** μ-CT 3D reconstruction. The obverse, the reverse and the enlarged interested area around the cranial raphe were shown. Hematoxylin and eosin (HE) staining and tartrate-resistant acid phosphatase (TRAP) staining for the Ti particle model **(C)** and PMMA model **(D)**. Determination of the area of TRAP-positive cells for the Ti particle model **(E)** and PMMA model **(F)** (^∗^*P* < 0.05; ^∗∗^*P* < 0.01). PO, periprosthesis osteolysis.

Bone resorbing activity of osteoclasts plays an important role in wear-particle-induced osteolysis. Previous studies showed that anion exchange inhibitors could repress the bone resorbing activity of osteoclasts thought regulating pHi (Rousselle and Heymann, 2002; Supanchart and Kornak, 2008). Regarding this matter, we wanted to investigate whether DIDS, an anion exchange inhibitor, could suppress wear-particle-induced bone loss *in vivo*. In the mouse calvaria model used in this study to explore the effects of DIDS on particle-induced osteolysis, Micro-CT scanning and 3D reconstruction revealed extensive *in vivo* bone resorption in the wear particles group that was observed as extensive surface erosion on the calvaria (wear particles vehicle), when compared with the negative control (PBS sham). However, treatment with DIDS suppressed wear particle-induced osteolysis in a dose-dependent manner, with bone resorption in the mice treated with a high concentration of DIDS being considerably lower than in mice treated with a low concentration of the compound (Figures 4A,B). Histological assessment confirmed that DIDS treatment protected against Ti- and PMMA-induced bone loss. HE staining revealed few osteolytic changes in sections from the sham group. By comparison, osteolysis had clearly occurred in the vehicle group, whereas the DIDS-treated groups exhibited reduced osteolysis (Figures 4C,D).

The leading cause of PO is osteoclast differentiation and activation promoted by wear particles (Goodman and Ma, 2010). Consistently with this fact, TRAP staining revealed an increase in the number of multinucleated osteoclasts at the injection site in the presence of wear particles, as indicated by the presence of osteoclasts lining the eroded bone surface. However, in both the low- and high-dose DIDS treatment groups, the osteoclast surface area was reduced (Figures 4C–F), indicating the blockage of wear-particle-induced bone loss partly via targeting osteoclast *in vivo.*

### Controlled Release of DIDS From CMs Effectively Strengthen the Inhibition Effect of DIDS on Wear Particle-Induced PO *in vivo*

**FIGURE 5:**
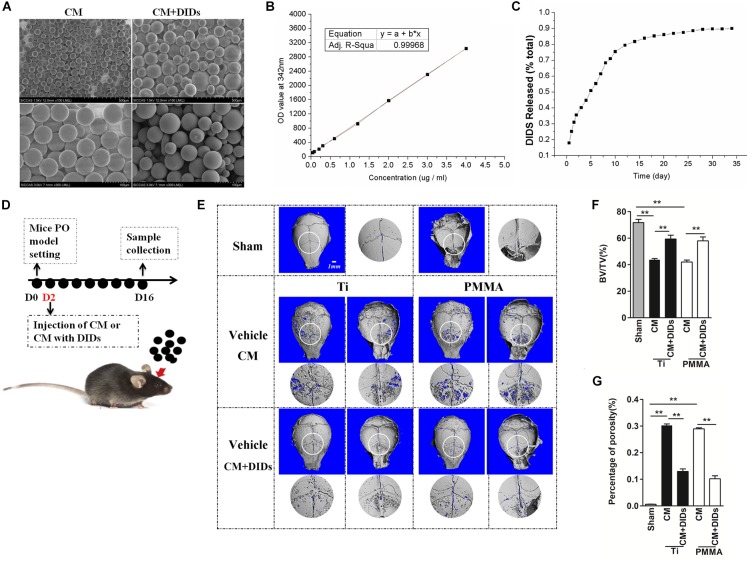
Therapeutic effect of chitosan microspheres (CMs) encapsulating DIDS on mice wear particle-induced osteolysis. **(A)** Scanning electron micrographs of CMs. **(B)** Standard curve of DIDS. **(C)** The control release ability of CMs. **(D)** Timeline of the experiment. **(E)** CMs encapsulating DIDS controlled wear particle-induced osteolysis demonstrated by micro-CT. **(F)** Bone volume against tissue volume (BV/TV), **(G)** the percentage of total porosity (%) of each sample was measured (^∗^*P* < 0.05; ^∗∗^*P* < 0.01).

As mentioned above, DIDS displayed satisfactory therapeutic effects in the mouse model. However, it is usually difficult to maintain an effective concentration of drugs, which hinders their further application. In order to elicit a sustaining effect, a strategy for controlled release of DIDS should be developed. CMs represent a useful tool for modified drug delivery, as their preparation is quite simple and they are useful for controlled drug release (Islam et al., 2012). In this study, we used CMs to encapsulate DIDS for controlled release *in vivo.* The CMs were spherical in shape with a mean size of approximately 50 μm (20–60 μM) with a smooth surface without cracks or wrinkles, as demonstrated by scanning electron micrographs (Figure 5A). The controlled release capacity of CMs was determined by measuring the OD value of the extracts at 342 nm (which is specific for DIDS). DIDS was released from the microspheres in a biphasic fashion, characterized by a fast release phase during the first 10 days followed by slower release on the remaining days (Figures 5B,C).

Chitosan microspheres encapsulating DIDS were injected into the OA joints 2 days after surgery, and subjects were then analyzed by microCT (Figure 5D). When crania were set for the PO model and further treated with CMs containing DIDS, they displayed markedly decreased osteolysis. BV/TV increased and the percentage of porosity decreased compared with crania treated with CMs with PBS (control group), thus indicating a delay in PO development (Figures 5E–G).

## Discussion

Periprosthesis osteolysis and subsequent aseptic loosening remains a major problem for joint arthroplasty failure (Yang et al., 2004). The mechanism between wear particles-induced and normal osteoclast differentiation may not be the same (Abbas et al., 2003). Ti and PMMA are common particles that appear in tissues around joint prostheses and contribute to osteolysis by stimulating osteoclast differentiation (Hirakawa et al., 1996; von Knoch et al., 2004; Baumann et al., 2005; Purdue et al., 2006). In this study, we used Ti and PMMA particles as a surrogate for wear debris to investigate role of SCL4A2, the osteoclast’s anion exchanger, in wear particle induced- osteoclastogenesis and bone resorption *in vitro* and possible treatment effect for PO by suppression of anion exchange *in vivo*. To our knowledge, our study is the first to illustrate the importance of anion exchange activities inhibition by DIDS in reducing wear particle-induced PO.

Bone resorption is the most important function of osteoclasts, which is the basis of wear particle induced-osteolysis. The first step in the resorption of bone by osteoclasts is polarization, which relies on the orderly arrangement of actin adhering to the bone surface to form a sealed area, with osteoclasts attached to the periphery to form a ruffled edge (Hartmann and Tabin, 2001). The ruffled edge forms a ring-shaped zone of bone absorption that is full of filaments, with lysosomes produced on the surface of the ruffled edge that secrete a large number of molecules, including carbonic anhydrase II, H^+^ ATPase, cathepsin K (CTSK). SLC4A2, which is an electroneutral Cl^-^/HCO_3_^-^ anion exchanger regulating the export of carbonate ions and import of chloride ions (Rousselle and Heymann, 2002; Bonar and Casey, 2008; Supanchart and Kornak, 2008; Alper, 2009; Parker, 2018). As such, SLC4A2 plays an important role in the regulation of intracellular pH during cell alkalinization, as well as controlling cell volume via Cl^-^ uptake (Rousselle and Heymann, 2002; Supanchart and Kornak, 2008). It has been recognized that SLC4A2 is upregulated during osteoclast differentiation, being a critical mediator of osteoclast differentiation and function (Wu et al., 2008). During the differentiation of osteoclasts induced by Ti and PMMA particles, the gene expression of *Slc4a2* gradually increased over time, indicative of a key role of SLC4A2 in wear particle-induced osteoclast differentiation. Furthermore, knockdown of *Slc4a2* gene expression inhibits osteoclast differentiation and function *in vitro*.

We also confirmed that SLC4A2 expression is dependent on the CN/NFAT pathway during wear-induced osteoclast differentiation. Specifically, the downregulation of *Slc4a2* expression also reduced the expression of *Nfatc1*, *Trap*, *Ctsk*, and *Ctr*, which are osteoclast-specific genes induced by a decrease in wear particles. Furthermore, a previous study reported that loss of SLC4A2 function in osteoclasts leads to abnormal actin belt formation, cell spreading and migration, indicative that maintenance of intracellular pH in osteoclasts through anion exchange regulates the actin superstructures required for bone resorption (Coury et al., 2013). If the actin ring of osteoclasts is dysplastic or damaged, osteoclasts are unable to exert their bone resorption and apoptosis effects (Kang et al., 2006). After SLC4A2 downregulation, the number of actin rings was significantly reduced for both Ti or PMMA groups. Additionally, Coury et al. (2013) found that SLC4A2 might regulate bone resorption and actin cytoskeleton organization in osteoclasts via calpain activity mediated the anion exchange-dependent maintenance of pHi. Therefore, we hypothesized that blocking SLC4A2 expression or function could significantly inhibit wear-induced osteoclast bone resorption activity, in part due to a disruption of the actin ring structure of osteoclasts.

Based on the above information, inactivation of SLC4A2 expression or function is probably a novel target for the treatment of particle-induced osteolysis, which was consistent with our *in vivo* experiments. DIDS has previously been shown to inhibit the basolateral Cl^-^/HCO_3_^-^ exchange activity of SLC4A2 in a dose-dependent way (Ramasamy et al., 2001). A previous study has reported on the benefits of DIDS in inhibiting apoptosis in myocardial ischemia caused by ischemia reperfusion and anti-tumor therapy (Wang et al., 2015). In our *in vivo* model, as DIDS is not a specific inhibitor of osteoclast anion exchange, we chose to apply topical drugs to avoid interference with other systems which could have biased our results. DIDS in our model significantly reduced wear particle-induced bone loss. Therefore, DIDS could be a promising agent for reducing the level of wear particle-induced osteolysis.

The difficulty in maintaining an effective concentration of drugs is well known and makes further application difficult. This warrants further investigation on developing a strategy of local delivery of DIDS for PO treatment. Local delivery of reagents will be more effective at targeting with less associated side effects. CMs have potential application for drug delivery systems because they are an inexpensive, biocompatible, biodegradable, and non-toxic natural polymer that enables the controlled release of many drugs (Jain et al., 2004; Kang et al., 2006). We found that controlled release of DIDS from CMs effectively strengthened the inhibitory effect of DIDS on wear particle-induced PO *in vivo*.

In summary, anion exchange mediated by SLC4A2, through its effect on the formation of the actin rings of osteoclasts, plays an important role in the differentiation and function of wear particle-induced osteoclasts. Thus, blocking the SLC4A2 expression or anion exchange function may provide new targets for the treatment of PO. Furthermore, the positive effects of DIDS in reducing the area of bone reabsorption in our animal model provide preliminary support for the plausible role of DIDS as therapeutic agents for the treatment of aseptic loosening or other osteoclastic-related osteolytic diseases.

## Author Contributions

FL, DY, and ZZ designed the research. CW and XL performed the research. RS prepared the ShRNAs for SLC4A2 and *in vitro* experiments. ZL and TT contributed new reagents or analytic tools. SY prepared the CMs. CW and YQ analyzed the data. CW wrote the paper.

## Conflict of Interest Statement

The authors declare that the research was conducted in the absence of any commercial or financial relationships that could be construed as a potential conflict of interest.
