# Corrigendum: Targeting Anion Exchange of Osteoclast, a New Strategy for Preventing Wear Particles Induced-Osteolysis

**DOI:** 10.3389/fphar.2019.01078

**Published:** 2019-09-23

**Authors:** Chuanlong Wu, Xuqiang Liu, Ruixin Sun, Yunhao Qin, Zhiqing Liu, Shengbing Yang, Tingting Tang, Zhenan Zhu, Degang Yu, Fengxiang Liu

**Affiliations:** ^1^Shanghai Key Laboratory of Orthopaedic Implants, Department of Orthopaedics, Ninth People’s Hospital, Shanghai Jiao Tong University School of Medicine, Shanghai, China; ^2^Department of Orthopaedics, Ruijin Hospital, Shanghai Jiao Tong University School of Medicine, Shanghai, China; ^3^Department of Orthopaedics, The First Affiliated Hospital, Nanchang University, Nanchang, China; ^4^State Key Laboratory of Oncogenes and Related Genes, Shanghai Cancer Institute, Renji Hospital, Shanghai Jiao Tong University School of Medicine, Shanghai, China; ^5^Department of Orthopaedics, Sixth People’s Hospital, Shanghai Jiao Tong University, Shanghai, China

**Keywords:** bone resorption, wear particle, osteoclast, SLC4A2, actin

In the original article, there was a mistake in **Figure 1** as published. **Figure 1C** did not correctly match the description in the figure legend. The corrected **Figure 1** appears below.

**Figure 1:**
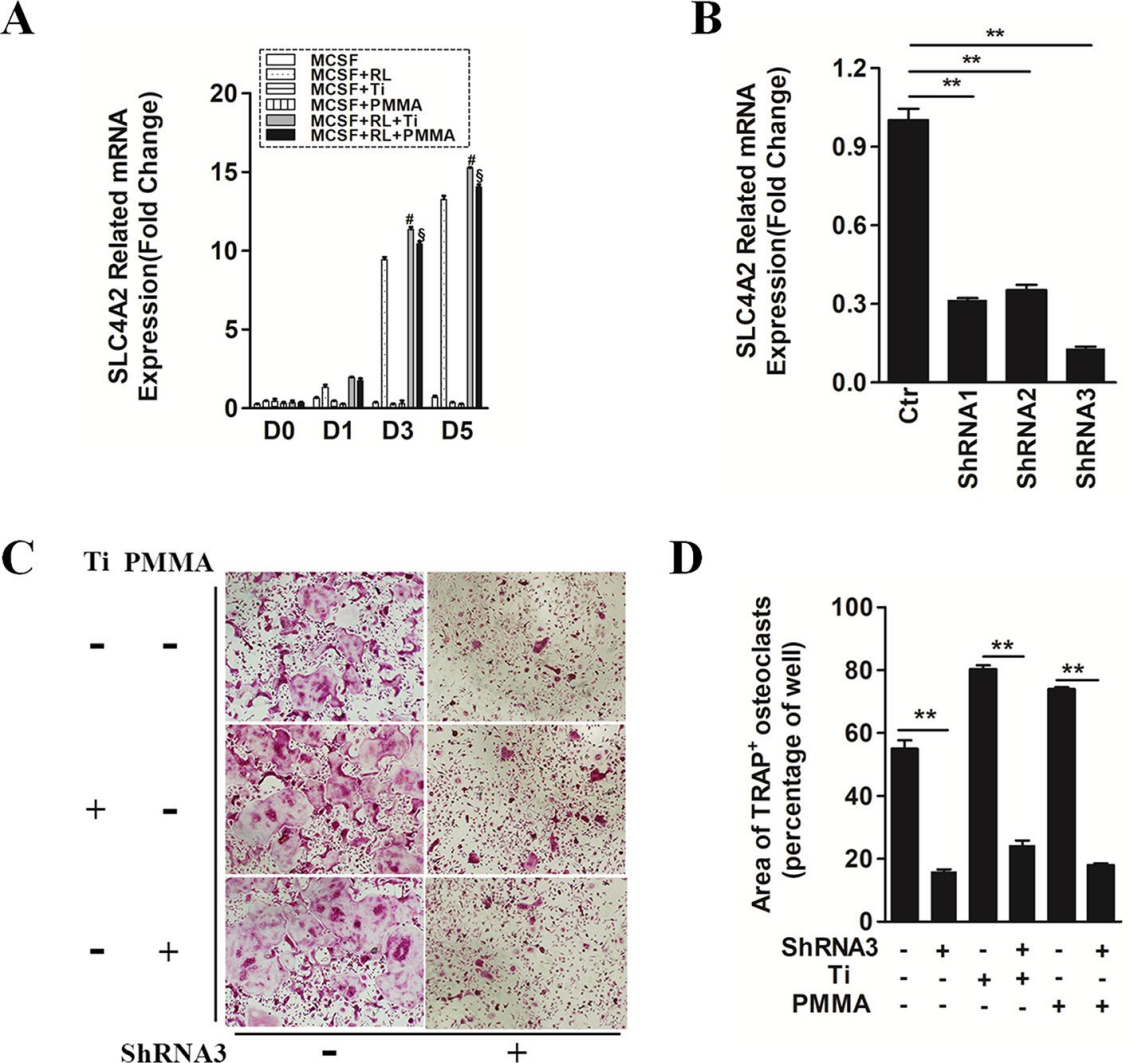
SLC4A2 plays an important role in wear particle-induced osteoclastogenesis. **(A)** Up-regulation of *Slc4a2* gene expression in the process of wear particle-induced osteoclastogenesis. Bone marrow-derived macrophages (BMM) were used [#, MCSF+RANKL(RL)+Ti vs. MCSF+RL, *P* < 0.01; §, MCSF+RL+PMMA vs. MCSF+RL, *P* < 0.01]. **(B)** Three different shRNA vectors targeting *Slc4a2*, with shRNA3 yielding the greatest reduction in *Slc4a2* mRNA. Effective knockdown of *Slc4a2* in BMM cells, at 48 h after transfection using *Slc4a2* shRNA1, shRNA2 and shRNA3, respectively. After transfection, cells were induced to differentiate into osteoclasts and harvested to examine *Slc4a2* expression using reverse transcription quantitative PCR (RT-qPCR). **(C)** Effect of knockdown of *Slc4a2*, using shRNA3, on wear particle-induced osteoclastogenesis *in vitro*. **(D)** The area of TRAP-positive cells, measured using ImageJ (*P < 0.05; **P < 0.01). At least three independent replicated of each experiment were conducted separately.

The authors apologize for this error and state that this does not change the scientific conclusions of the article in any way. The original article has been updated.

